# Trusted Professional Multi-Agency Transitions for Young People Facing Multiple Disadvantage – Learning from Co-Production by a Third Sector Partner in the Plymouth Alliance, UK

**DOI:** 10.5334/ijic.9055

**Published:** 2025-07-21

**Authors:** Gemma Doyle, Sean Mitchell, Sue Hawley, Katy Krysiak, Felix Gradinger

**Affiliations:** 1 The Zone, Plymouth, UK, Registered Charity No. 1051757; Company Registration No. 3140076; https://www.thezoneplymouth.co.uk/; 2 Changing Futures Plymouth, Community Connections, Plymouth City Council, UK; https://theplymouthalliance.co.uk/changing-futures; 3 National Institute of Health and Care Research (NIHR) Plymouth Health Determinants Research Collaboration (HDRC), Public Health, Plymouth City Council UK; https://www.plymouth.gov.uk/plymouth-health-determinants-research-collaboration-phdrc; 4 Improving Lives Plymouth, Plymouth, UK, Registered Charity No. 1066776; Private Limited Company by guarantee without share capital use of ‘Limited’ exemption Reg No 2610208; https://www.improvinglivesplymouth.org.uk/; 5 Community and Primary Care Research Group, Faculty of Health, University of Plymouth, UK; https://www.plymouth.ac.uk/research/primarycare

**Keywords:** transitions, young people, multiple and complex needs, trauma, childhood adversity, broader determinants of health, vulnerable/disadvantaged

## Abstract

**Introduction:**

This case study provides practice-based reflections on challenges and potential solutions for young people with multiple disadvantages across housing, substance misuse, mental health, criminal justice, and domestic abuse systems, informed by 4 local principles: trauma informed, learning based, an alliance commissioning ethos, and workforce development.

**Description:**

To improve the current experiences of 17–25-year-olds in service transition iterative insights drew from networking staff across sectors, clinical audit and following live cases, and appreciative enquiries with young people. This was conducted by a practitioner researcher in a local Young Person’s charity and was supported by peer researchers with lived experience and embedded researchers-in-residence.

**Discussion:**

This describes the scale of the challenge where compound need and intersectional disadvantage, wider determinants, complex pathways, and public and third sector service systems collide. Relational practices were tested to support navigating system challenges, better tailor to young people’s abilities and needs and improve integrated care partnership working and workforce development.

**Conclusion:**

Plymouth has a history of integration with the Alliance for Complex Needs. Context and localised solutions matter for integrating care, yet remain underreported especially for underserved, and marginalised young people and using whole systems approaches co-produced with the third sector. Investment into academia-practice partnerships is crucial to make learning portable.

## Introduction

The 2008 findings of the *WHO Commission on Social Determinants of Health* [1] are relevant today as, according to the *United Nations (UN)*, 71 percent of the world’s population live in countries where inequality has grown [2]. The related 2015 *Lancet* series captures the challenge of social disadvantage with well-documented effects on developing brains and limiting children’s intellectual and social development and a clear ‘social gradient’; i.e. the higher the social position of families the more children flourish and the better they score on all development measures [3].

As of October 2024, 36% of children in the United Kingdom (UK) were living in poverty [4]. This was conservatively estimated to give rise in direct and indirect costs to £40 billion a year by 2027 [5]. Research on the cumulative, multi-factorial and devastating effects of Adverse Childhood Experiences (ACEs), is particularly well documented in literature reviews around physical [6] and mental health [7], and less so for other related public services [8,9], the criminal justice system [10], and the wider structural determinants.

What this may mean to families re-living ‘inter-generational cycles of trauma’ [11], is that the compartmentalization of compound and colliding needs often fails our most vulnerable young people by the very design of public services siloed through New Public Management (NPM) [12], while contrasting with less transactional ‘third sector’ (for ease we chose this term, in the UK variously referred to as the Voluntary, Community, Faith, and Social Enterprise-VCFSE sector, elsewhere: ‘not-for-profit’, ‘voluntary welfare organisations’, etc) approaches increasingly picking up the slack, responsibility and risk [13].

This way of organizing services can be found to add little value through bouncing people between differing professional responsibilities and statutory obligations (i.e. through repeat assessments, risk and eligibility thresholds, handovers, waiting lists, rationing of services under austerity measures), thereby creating additional, costly and preventable ‘failure demand’ (John Seddon), often reactive at crisis points only [14]. All the while this can offer a poor and fragmented service experience [15], which can feed mutual mistrust [16] between a vicariously traumatized workforce and often re-traumatized young people [17].

### Lack of a comprehensive academic evidence base

This fragmentation is mirrored in terms of academic attention given to the complex and ‘wicked problem’ (Keith Grint) of transitions from adolescence to adulthood for young people with multiple disadvantages, who are often relying on different public services across different sectors, and which does not lend itself easily to linear, controlled and reductionist research approaches. Most of the evidence we found through non-systematic searches of literature reviews (only) appears to be looking at symptoms rather than addressing underlying causes and appears top-heavy following a hierarchy of evidence from health down to other, lesser-researched public services. This is exemplified with the finding that the most cited research is being conducted on vertical transitions (rather than horizontal, functional, clinical or service integration [18]) within health settings [19,20,21], and or mental health services [22,23,24]. Respectively, in social care research the academic focus appears to be on statutory obligations and transitional issues between children and adult social care with a focus on transitions of care leavers and care-experienced young people [25,26]. More rarely the literature reviewers’ perspectives are flipped more functionally by looking at particular outcomes like employability for complex needs, more holistically defined to include health and social exclusion issues, in one review we found [27].

### Defining integration, integrated care, multi-agency working and multiple disadvantage

A large European program of research concluded in 2017 that inter-agency partnerships (e.g., health care, youth care, social work, education, welfare) have become increasingly recognized as important for policy to support children and families [28]. This report defines ‘multi-agency’ working as: more than one agency working with a client, not necessarily jointly, which can be concurrent or sequential, with joint planning. This conceptualisation is then contrasted with consecutively deeper levels of integration with: ‘inter-agency working’ – planned and formal way at strategic or operational level; ‘joined-up working’ – coordinated planning inclusive of multiple policies and agency practices; and ‘integrated-working’ – defined as everyone supporting children and families together effectively, putting the child at the centre to meet their needs, through formalized collaboration and coordination between agencies.

The most relevant integrative, qualitative literature review we found explores social work practices for adolescents and young adults with complex needs based on work in Sweden [29], summarizing that the concept is “applied to a fairly heterogeneous group, often recipients of long-term, but not very successful support within the welfare system”. Mirroring some of the siloing from the academic literature above, the paper helpfully lists a range of definitions offered, largely through a service lens and which vary in tone and whether seeking to employ more strengths-based or neutral language (i.e. range from: “especially disadvantaged people”; “people presenting challenges to services”; “multiple interconnected needs that span medical and social issues”; “situations where young people are burdened by multiple and co-occurring problems”; “multiple service-using youth”).

We agree with the authors that any definition needs to be flexible enough to include nuance and variation while also, and crucially, considering the complexity of the health, care and wider public service system which, in its commodifying logic [30], is seeking to standardise, label and categorise people as ‘complex’ to control and routinise care. Any definition is therefore imperfect and as this paper seeks to show, what might be more important are the underlying values and principles that inform how services and young people can better work together in a bespoke way that builds on what matters to each individual person and circumstances. In exploration with peer researchers and partners this programme chose to use the concept of ‘multiple disadvantage’ as it avoids the deficit-based term ‘need’, seeks to take a de-personalised and less judgemental view of lived experiences (see social model of disability), and may be more inclusive of ‘multiple’, compounding and colliding scenarios presenting at any given time in a person’s life.

Academic-practice partnership informed by Human Learning Systems (HLS) [31] and ‘system stewardship’ using an embedded researcher-in-residence approach may offer creative solutions in context. In the UK this may be happening in various forms of embedding learning via recently funded National Institute of Health and Care Research (NIHR) Health Determinants Research Collaborations (HDRC) [32], hosted by public health departments across 30 local government sites in the UK. They may show the way of co-producing whole-systems solutions systematically within regional contexts [33], to facilitate more immediate improvement (do-know-gap), rather than waiting for an average of 17 years for traditional evidence creation to feed into policy decisions (know-do-gap) [34]. HLS approaches entail the parity of knowledge creation between lived (peer researchers), practice-based (practitioner researchers), and academic learning (embedded researchers-in-residence; https://www.embeddedresearch.org.uk/; accessed 27.05.25). They emphasize the need to progress towards the more systematic collection of relevant and timely formal and informal knowledge to inform and drive improvement in service delivery, change the culture of diverse and disparate organizations and stakeholders towards more reflective and iterative learning practice, develop the skills to achieve this, and translate better understanding of what works, turning interventions into sustainable improved practice.

This Integrated Care Case (ICC) aims to report on the potential of an action learning approach [35] to developing existing working practices between cross-sector, multiagency professionals working with young people experiencing multiple disadvantages. This paper seeks to report on: a small-scale prototype that is currently and still evolving (integrated care), which is happening in parts of the system that may be more integrated (through alliance commissioning in the City). It seeks to provide a humble example of how the third sector is constantly innovating, experimenting and learning by any means available and invites the academic community to engage with such approaches.

This practice-based report can be seen as an implementation research output about context and potential future practice, with further impacts and detailed findings reported in future. This is not ‘big R’ research and instead seeks to capture our collective practice-based and academic reflection and describing with adequate detail the innovative middle ground between relevance and rigor of a mixed practice of clinical audit, service development/improvement/evaluation. Qualitative, quantitative and experiential insights were gathered from an array of small iterative pieces of work that led to a local test and learn pilot project of a ‘trusted professional’ approach to support the transition to adult services in multiple systems, for 17–25-year-old young people in the City of Plymouth, in the Southwest Peninsula of the UK.

## Setting

This work is part of ‘The Changing Futures’ Programme (https://www.gov.uk/government/collections/changing-futures; accessed 27.05.25) since the end of 2021 and is seeking to play a significant role in system change. Underpinning this purpose are four principles: 1) co-produced; 2) trauma-informed; 3) learning based; 4) alliance ethos.

One of the projects reporting here evolved from innovation based out of The Zone (https://www.thezoneplymouth.co.uk/; accessed 27.05.25). The Zone is a youth enquiry service and a registered local charity that offers a variety of vital, free support including mental health, housing advocacy and sexual health services. It is the ‘gateway’ for 5,000 young people annually in the City. Like many other third sector ‘one-stop-shops’ in the UK [36], it seeks to engage people in a relational and responsive way and helping them navigate busy lives and the complexity of public services.

The Zone is one of seven members of the ‘Plymouth Alliance’ for Complex Needs. The Plymouth Alliance is a collectively commissioned contract awarded by the City Council for the provision of support in relation to homelessness and support needs around substance misuse, mental health, offending and risk of exploitation. The ‘Alliance Ethos’ (https://www.humanlearning.systems/uploads/Plymouth%20Alliance.pdf; accessed 27.05.25) is not only referring to the contractual obligations for multi-agency working, but rather all the relevant services supporting people with multiple disadvantages that form ‘the system’, to remove barriers caused by pre-existing contracts and enhance partnerships and collaborations across the City [37,38].

Both Plymouth and Westminster (https://www.westminster.gov.uk/changing-futures/specialist-team; accessed 27.05.25), acknowledged youth transitions as an area requiring a change due to being in the top 25 local authorities in the UK benchmarked with a high prevalence of three indicators of multiple disadvantage (Homelessness, Substance Misuse, Offending behaviours) [39].

## Implementation approach

The table below gives an overview summarizing the process, to serve as a portable blueprint. It showcases the iterative and necessarily pragmatic nature of working with what was found in context and given resource constraints, going from broad mapping and exploration of service and service user journey maps, on to explorations of lived and professional experience, resulting in a small-scale pilot of the trusted professional approach (Table 1).

**Table 1:** Overview of implementation process and key learning.


STAGES AND TIMELINE OF THE WORK	ROLES	APPROACH	OUTPUTS	ITERATION/IMPACT

(3.3) Fact-finding; Learning by listening to local services Aug 2022 – Mar 2023	Conducted by: GD	Semi-structured interviews and service mapping with 25 services.	Service data for system mapping and identifying overarching messages.	Identify gaps/demands, workforce needs; outcome measures; risks.

(3.4) Clinical Audit; Reviewing records of service transitions Oct – Dec 2022	Conducted by: GD Supervised by: SM	40 young people’s interactions within homelessness services; 20 with advocacy/20 without.	Journey mapping 6 months of case files; auditing information and sharing agreements.	Face-to-face meetings improved understanding and coordination

(3.5) Peer Researcher Placements; Exploring young people’s current lived experience Feb – Dec 2023	Conducted by: SH Supervised by: GD & SM	Hosting two peer researchers at The Zone coproducing ‘Your Story’ project using Appreciative Enquiry (AE).	Collecting stories from 4 consenting young people for sense-making and journey mapping with services.	Emerging themes; picking up on trends; marrying above findings to develop Transitions Matrix forum.

(3.6) Transitions Matrix; Facilitate cross-sector, multi-agency networking and innovation forum (ongoing)	Co-facilitated by: GD	Co-facilitate a monthly forum for service introductions, updates, networking and innovation with Children and Adolescents Mental Health Services (CAMHS) transitions team.	18 forums to date, each with 25-40 in attendance, information sharing, including training and 48 service presentations.	New partnership developments, from opportunities to network and for peer support and sharing best practice. 180 people on mailing list from 80+ services.

(3.7) Live case Test and Learn Pilot of the TP Approach Nov 23 – Mar 24	Practice pilot: 4 x Trusted Professionals and other supporting professionals Cases observed by: GD, SM & SH Interviews with TP’s conducted by: SH Learning Groups Co-facilitated by: KK, SH, FG Write up: ALL Authors	Following 6 cases of young people in the homelessness system in real-time with identified TP; observations of multi-agency meetings.	Monthly update and cost-benefit demonstrated to strategic leaders and 3 interactive learning groups/workshops with involved professionals.	Positive practice shared and disseminated locally and nationally through evaluation reports. Ripples to other HDRC pilot work.

(3.8) Reflective Practice; Keeping a reflexive journal throughout.	Reflexive journal: GD, SH Debriefs between: GD & SM/SH/FG	Ongoing reflexive diaries and documentation; de-briefs with peer researchers and colleagues.	Learning from peer researcher placements; Secondment to HDRC.	Building capacity; research and learning champion for third sector across City.


This table illustrates the iterations of implementation since 2021. A more detailed evaluation report can be available on request. Given time and space constraints, we also intend to analyze and report these findings in more detail and more robustly elsewhere.

(3.3) Factfinding involved conducting informal conversations with 25 Plymouth services, discussing service remit, capacity, demand and reflections on current transitions, challenges and gaps identified in local systems. As many relevant local services as possible were contacted to speak about transitions. Through snowballing and convenience sampling 25 conversations took place.

(3.4) Internal audit involved understanding and mapping current transition journeys from youth to adult homelessness support services, gathered from case, meeting and assessment notes.

(3.5) The ‘Your Story’ project aspired to have one Appreciative Enquiry conversation a month over six months with at least six young people. Due to the compound nature of multiple disadvantages such engagement proved not viable for most participants. Four YP took part, three young people had one conversation with a peer researcher and one YP had three conversations. Inclusion was based on the live experience of homelessness and multiple disadvantage. The project was advertised through The Zone and shared with other local youth services. The young people that came forward either had an existing trusting relationship with a worker at the Zone or a service that partners with the Zone. Exclusion criteria included anyone over the age of 25.

Early themes identified include:

- Intergenerational challenges – repeated homelessness
- Lack of continuity and consistency in care
- Not being heard
- Being guided – need for bespoke support
- Being held and being safe
- Having purpose or meaning
- Being loved/understood
- Having trust with at least one professional

(3.6) Disjointed services was an emergent message from both services and young people. To nurture service connections the ‘Transitions Matrix’ was developed. This is a 1.5-hour monthly face-to-face meet, network and innovation forum for cross-sector services working with transitional-aged young people (16–25 years old, as per CAMHS transitions guidelines). This forum is co-facilitated with the local Child and Adolescent Mental Health Services (CAMHS) transitions team and provides opportunity for service introductions, networking and sharing information on events, training, and funding.

(3.7) The TP pilot project included attendance at youth homelessness intervention meetings to identify cases, and observing already existing Multi-agency Team (MAT) meetings to document developments and partnership working, cost mapping the developments for strategic leaders, and running learning groups with professionals involved in the final three months. Each stage involved:

- learning by doing, exploring what adaptations to the system are possible
- following live cases as they developed, how collaboration can be aided, and barriers overcome
- evaluating and adapting to changes in real-time,
- involving the professionals in the changes they want to see, complemented by interviews with TP’s,
- discussion with city-wide services, making cost-benefit arguments, and creating a community of practice
- ‘Your Story’ young people’s stories informing the progress.

(3.8) Constant reflective practice supported learning and one iteration leading to another, supporting peer-researcher placements, development of Appreciative Enquiry with young people, facilitating networking in the Transitions Matrix and learning groups during the TP pilot.

## The TP Approach: Assumptions of potential impact

A trusted professional (TP) can be defined as any worker, in any sector, service or system, whom a YP has a trusting relationship with. They are enabled to listen to and understand the issues a YP is asking for support for and pulls in the right professionals to address surrounding issues. The TP approach and the principle of dedicated key workers is not a new idea (CF Northumbria have been working on the ‘liberated method’ with adults for years [40], and key-worker roles are written into recent safeguarding statutes and policy nationally) [41]. Consequently, this approach was devised to build on trust between professionals themselves and with young people and in a localised context.

Instead of relying on largely unapplicable, siloed academic literature, it is notable that this project drew inspiration largely from learning developed by various central or local government departments, think tanks, third sector organisations and charitable funders (sometimes working in partnership with universities), who offer a plethora of relevant evidence, and which validate our approach and learning (see appendix for full list of these).

**Figure 1:**
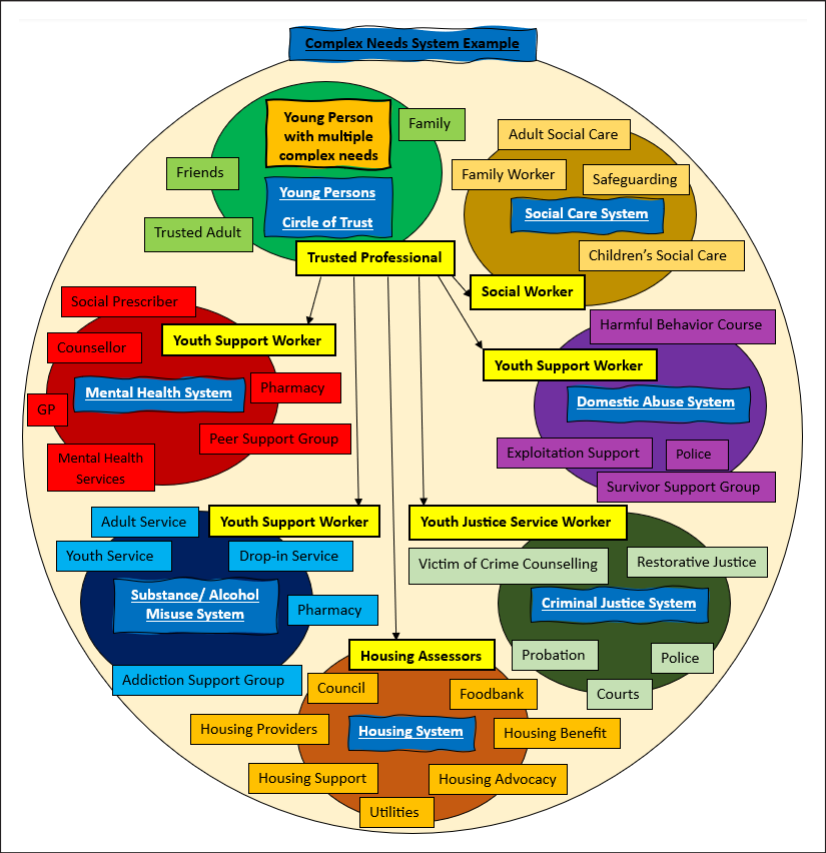
Mapping of local services and TP pulling support.

Our application of this learning locally shows young people experiencing multiple disadvantages may struggle to engage with multiple services at the same time, but the city offers a multitude of dedicated cross-sector, multi-disciplinary professionals who hold trusting relationships with young people. The TP pilot demonstrated that given time and flexibility these professionals might learn to trust each other, and truly understand a YP, their support needs and what matters to them. To avoid duplication and young people repeating their stories, a TP has the potential to act as a conduit for YP to other services and support them in developing trusting relationships with other appropriate services. See a mapping of local service configurations in Figure 1.

**Figure 2:**
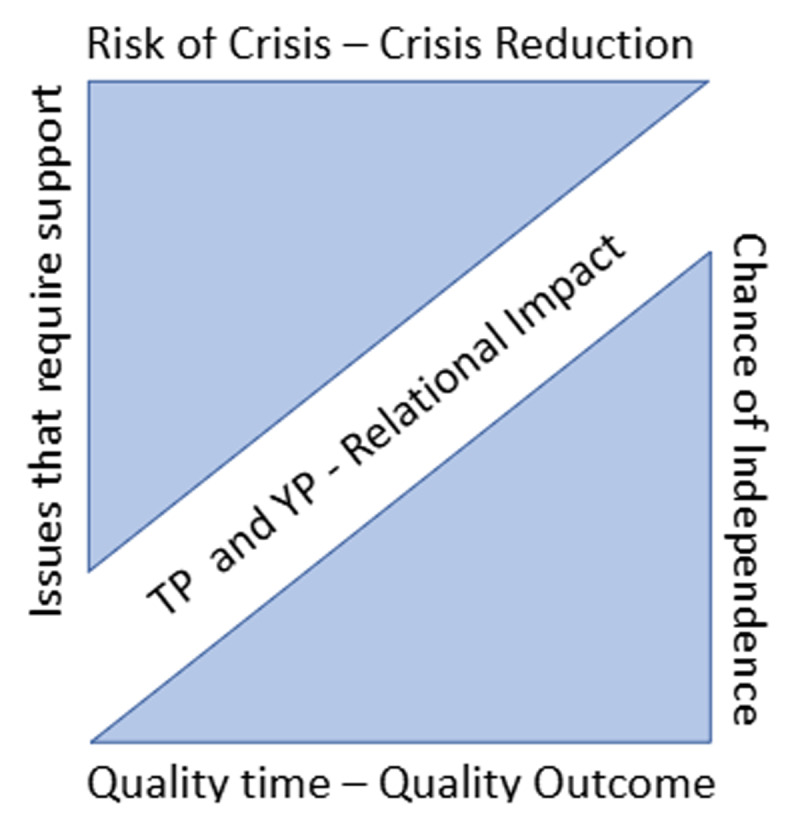
Visualization of Trusted Professional relational impact hypothesis.

For the TP pilot it was hypothesized that the relational impact of quality time to address issues that require support would reduce the risk of reaching a costly crisis point, and increase the chance of independent support management in adulthood (Figure 2).

## Process and roles

The work was conducted by the Partnership Development Coordinator for youth transitions (GD) at The Zone, funded for 3 days a week from August 2021 – July 2024 by Changing Futures Plymouth, then seconded for a 9-month period to the Health Determinants Research Collaboration from August 2024. They were supported by the operations manager at The Zone (SM). Both individuals had gained experience as practitioners in homelessness and youth services, building evaluation and research skills with an ambition to become practitioner researchers (later supported with training, supervision and this report by academic FG). This ICC reflects on the learning element and iterations of a co-produced process involving open, appreciative enquiry [42] and lived (SH) and professional experience. Thus the service development and co-production (practice-based learning) in early phases of the project, was complemented by academic learning enhanced in the later and currently ongoing partnership.

The learning reported here is loosely informed by a developmental evaluation approach [43], with each emergent iteration of collective sense making feeding into the next. Through extensive networking, the project largely relied on opportunistic convenience and snowball sampling to pull in stories from young people, staff and managers, using largely qualitative with some quantitative process and case tracking data (a form of clinical audit). Each stage of the collective sensemaking process was participatory in nature and done with triangulation resulting from peer researcher support, managers and colleagues as well as the staff participants.

### The role of peer-, practitioner- and embedded researchers

Through Changing Futures Plymouth, 12 individuals with lived/living experiences were recruited via a trauma-informed process (https://shows.acast.com/plymouth-changing-futures-podcast/episodes/ilp-the-peer-researchers; accessed 27.05.25), to be peer researchers within Improving Lives Plymouth (a Health & Wellbeing charity), who would offer them tailored support into and progression within and post placement across the system. This formed the Peer Research Network, which has had success in terms of high staff retention and satisfaction, as well as significant local and national reach, and led to the development of a Trauma-Informed Approach to Recruitment toolkit.

Each peer researcher was uniquely placed to embed themselves within local services and systems, develop trusting connections with, and gain valuable insights into the current experiences of individuals facing multiple disadvantages within them. Two youth-focused Peer Researchers with expertise-by-experience in disadvantaged young people were placed within The Zone. Amongst the work co-produced within this placement was the ‘Your Story’ project, which recruited young people passionate about having their experiences heard to create lasting change (see overview table above, and findings to be reported in future).

As renumeration for sharing valuable and vulnerable lived experience, young people were offered £25 Love2shop vouchers for each conversation. This project adopted a youth focused and trauma-informed approach. The recruitment poster/process, information sheet, consent form and debrief were all co-developed and informed by the team’s previous experience of engaging with young people living complex lives (see appendix for study materials). The peer researchers were responsible for building trust with potential participants, conducting narrative appreciative enquiry interviews, debriefing, transcriptions, early coding/theme identification and journey mapping.

For the safety and wellbeing of the vulnerable cohort of young people, each person was already known to The Zone, within which they were recruited for assurance of support. The original intention was for them to attend monthly over a 6-month period to relay month by month updates on their current living situation. However, in practice, despite debrief and in-house on-going support offered at The Zone, pre and post interview, young people experiencing on-going complexity in their living experience felt unable to engage with multiple sessions. The team were led by young people’s choices to avoid traumatization, which can often be experienced when repeatedly sharing difficult experiences. We did know a lot of their situations due to them working with professionals within or worked closely with the Zone and from the stories they shared with us, none of these young people were without indicators of significant number of ACE’s.

Following this placement, the two peer researchers moved on to other host organisations, however one remained involved (SH) through the development and implementation of the Trusted Professional pilot, in the learning groups, observing Multi-Agency Teams meetings and conducting interviews with the professionals involved. TP’s were selected due to their close working relationship with YP identified within the first 1–3 months of the pilot from the YHIP (Young Persons Homelessness Intervention Prevention programme), with meetings, held weekly, hosted by the Operations Manager at The Zone at the time (SM). Any other professionals working in any system who has experience working with young people enough to have developed a trusting relationship with the young people were included. This was initially coordinated by SM & GD and other city service actors in attendance and grew into an organic, self-organising community of practice (informed by some of the existing evidence listed in the appendix). Changing Futures Plymouth project support officer (KK) was instrumental in journey mapping some of the experiences collected from the ‘your story’ project with wider services, and supporting the TP pilot and learning groups. The learning groups introduced this way of working, as did informal coversations and attendance of SM and/or GD in existing Multi-disciplinary meetings to share local service expertise where relevant. TP’s identified also had support to be involved from their managers. This built on a system of pulling in collective knowledge already held by professionals in the city. This meant wider projects within the programme could be involved in the pilot’s delivery and future iterations. Other than calling Multidisciplinary Team Meetings ad hoc, there were no protocols, and business as usual was partly suspended and learning from live cases was generated opportunistically as they emerged and through the learning groups. TP’s and others involved identified what works and what does not, where the systems can have flexibility and where there are blockages. The focus was culture change with the hope of informing future process changes. Communication was fueled by practice knowledge, professional curiosity and non-judgemental attitudes towards creative solutions. Case responsibility and actions were shared and agreed as a group. Coordination and communication through regular meetings (rather than endless emails) may have saved time and ensured collective understanding. During the TP pilot, an HDRC embedded researcher-in-residence (FG) came onboard to support the learning to date and mentor and train the practitioner researchers and peer researchers.

## Learning from the TP pilot: limitations, further improvement and development

Supporting a YP through a TP, although showing traction and some emerging impacts, is not a recognised standard practice yet. Current New Public Management system design and delivery appear to create a systemic barrier which makes it challenging to justify and prove the value of the TP support provided. Due to many factors including time-limited support, caseload capacity, understaffing, contract provision etc., there can be a push approach: pushing out referrals or diverting a YP to other services to address other needs. This may be due to a number of barriers including: policy, capacity, and handovers, and may lead to poorer outcomes for young people and further financial constraints on the system. This may fail to account for the impact of trauma, leaving many young people without the tools or knowledge of services and systems.

While using peer researchers, the process has not yet involved the use of peer support workers more explicitly, as evidenced to be of effective and trusted help elsewhere [44]. We are also aware that the emphasis on the ‘trusted professional’ might be misconstrued to indicate a predominating service lens and taking an ‘adultist’ position, i.e. the inherent belief system that adults know best [45]. Furthermore, the direct involvement of young people in Multi-agency team (MAT) meetings was not yet explored systematically. However, one YP within in the pilot agreed to be supported to attend a MAT meeting. The informal feedback from those attending was that the conversations were more compassionate and conducive to the YP. This illustrates the potential to offer this choice when scaling up. Existing evidence of mediation through family group conferencing/decision-making suggests that involving a YP and their wider family and support networks could be a useful adjunct to this approach [46].

While this was not possible to evidence more robustly, young people, TP’s and other professionals were observed to fight for better outcomes, with some evidence of buy-in and support by wider city services and management in principle. The findings from running learning groups around multi-agency working, highlighted a significant need for more relational working and increasing existing appetite from professionals to adopt more individually tailored practice.

### Direct impacts on YP

One demonstration of impact was observed in the TP pilot. We saw that taking the time to understand and address unmet needs early in a YP’s support journey made a significant difference to housing outcomes. This included conducting a neurodiversity screening, invoking care act advocacy and a psychologist assessment of YP’s readiness for independent living. The implementation of these additional support measures early on, meant this YP’s unmet needs were understood by those assessing housing needs. This case resulted in a YP being suitably accommodated in specialist supported accommodation, where it’s believed they will only need to be in for one year to develop the skills required for long term independent living. This may have prevented unsuitable, unsupported placements with unmanageable tenancies that could have resulted in this YP re-cycling through the adult homelessness system.

### Professional and multi-agency collaboration

Addressing initial scepticism we learned a TP might not automatically involve more work as other professionals are still actively involved in the work. This may have resulted in an increase in trust between professionals through a practice of shared responsibility, improving relationships and partnerships, and overall increasing an appetite for positive risk-taking. Those involved might have been enabled to slow down to reflect with trusted peers on the best course of more coordinated and anticipatory care planning. This may contrast with standardised, reactive and routine responses to rush in to ‘fix’, duplicating work, siloing, and reverting to professional and statutory boundaries. Apart from mutual role-blurring and upskilling, amongst others this has also resulted in some new partnership and colocation arrangements with cross sector services.

### System and policy-level impacts

Wider impact may have come from the evidence base we have provided for system stewards in their role as practitioner- and/or peer researchers. The rich insights gathered for the ‘Your Story’ project and TP pilot were facilitated by true empathy and genuine compassion created in the system through living each other’s experiences (rather than lived experience being a token product).

Ripples from the work are still emerging, including integration with the family hub trailblazers (https://www.humanlearning.systems/uploads/PlymouthFamilyHubs.pdf; accessed 27.0.25), the Homeless Prevention Board, and developing a system wide adaptation of the ‘Team around me’ approach [47]. Changing Futures Plymouth is funding the initial training of 1–200 professionals from across the systems locally and The Plymouth Alliance & Devon Mental Health Alliance will be embedding this approach, with training workshops completed from Feb-May 2025. The ongoing iteration of this work is the current HDRC project, which includes testing the TP with tenancy support officers in the largest social housing provider in the city (https://www.plymouthcommunityhomes.co.uk/; accessed 27.05.25).

Practice Implications

- Trust between CYP and professionals, and between professionals: Co-production with YP is crucial to emphasise the key principles like ‘no wrong front door’ and ‘telling my story once’, to facilitate pull vs push approaches between professionals for earlier prevention of costly and siloed crisis response in fragmented public service systems.
- Best TP practice could involve YP nominating their own TP using a simple ‘circle of trust exercise’. Other models (e.g., Peterborough and Cambridge) allowed any trusted individual (not just professionals) supported by more resource and infrastructure, though it was tested with adults only (https://cambridgeshireinsight.org.uk/housing/changing-futures-cp/the-trusted-person-approach/; accessed 27.05.25).
- Recommendations for workforce development include developing specific training materials (https://welshwomensaid.org.uk/change-that-lasts/trusted-professional/; accessed 27.05.25). Appropriate supervision and support and for the TP’s to attend and running learning groups/reflective practice, alongside working on live cases would be beneficial in ensuring the model’s success.

Policy Implications

- The potential of channelling YP voices through living each other’s experiences through co-production and peer researchers (bottom up), building on cross-sector workforce wanting to do right by people (middle out) and changing culture of working practices through alliance commissioning with an attempt to spread and scale a whole systems approach (top-down).
- Larger, systemic paradigm shifts are needed, utilising the application of ‘proportionate universalism’, suggesting that ‘actions should be universal, but with an intensity and scale that is proportional to the level of disadvantage (61) may provide nuance in distinguishing between intentions, outputs and outcomes of a policy.
- Ongoing multidisciplinary dialogue is required, which may be guided by Harvard’s Human Flourishing Program (62) which aims to widen the view of what constitutes our virtue and intentions as human beings.

Research Implications

- An interdisciplinary research agenda covering horizontal, vertical and functional aspects is needed to shift from ‘multi-agency’, to ‘inter-agency working’, to ‘joined-up working’ and ultimately ‘integrated-working’ – where every worker across the system is collaborating and supporting children and families together, through more formalized collaboration and coordination between agencies (55).
- Potential impacts to consider in future research may include considerations of what matters to YP and process/experience outcomes (person-centered, goal-based, distance-travelled); as well as staff outcomes like improved communication, job satisfaction, data sharing, youth awareness, consideration of better YP involvement and wider preventative impacts (illustrated through qualitative journey maps, and quantitative theographs costing service activity over time).
- Stories collected from young women indicated that they may disappear more frequently than young men from service view at age 18 and come back more traumatised, and in more entrenched cycles of trauma. This highlights the gendered intersectionality at play around the homelessness and Violence Against Women and Girls (VAWG) agenda in particular (https://centrepoint.org.uk/sites/default/files/2023-04/In-her-shoes-young-womens-experiences-of-homelessness.pdf; accessed 27.05.25).

## Discussion – Compare and Contrast with international, academic literature

This practice-based report sits well within scarce qualitative research exploring ‘cross-systems’ transitions issues documented from young people themselves who have experienced homelessness, had children welfare systems involvement and educational challenges, and emphasising what actually matters to them in terms of goals, motivations, and aspirations, see this US study [48]. Further qualitative evidence from the US underlines the real risk of disengagement from transitioning in mental health services [49], emphasising that trust has to be earned through continuity provided over time and responding in a timely and bespoke manner. A Canadian literature review on ‘youth friendliness’ of mental health and substance use services stresses the importance of giving voice to young people and embed this in shaping all parts of the engagement including environment, policies, and treatments [50], as we have attempted to do. Decades of evidence around assertive outreach, open access youth work from Germany and wider Europe also emphasise these points, with interesting recent work in Germany developing social media ‘street work’ (https://european-social-fund-plus.ec.europa.eu/en/projects/social-media-streetwork-reaching-out-marginalised-people; accessed 27.05.25).

From the professional perspective recent evidence from the US criminal justice system highlights the discrepancy of professional versus young people perspectives and the importance of mediating between them through relational approaches [51].

Further qualitative research in Ireland highlights the cross-sector focus on managing risk of ‘complex youths’, rather than working with them in a rights- and strengths-based manner [52]. The same study suggested to introduce reflexivity of the workforce within multi-agency settings, which is something this project has started, and which was welcome by TPs attending the real-time Multi agency team meetings, related learning sessions, and the more informal peer support provided by the Transition Matrix meetings. This provides an argument to offer more support for the under-served, low paid, unregulated workforce, particularly in the third and other sectors, who are often more exposed to vicarious trauma. Contrastingly, the TP approach also demonstrated the flexibility available to this workforce for a higher tolerance and adaptability meeting complex needs, with the third sector teaching the art of the possible.

While we have found similar reports (see those listed in the appendix), the only robust and academic account we found through non-systematic literature searches was a relevant study following 166 Canadian multiple service using youth (at least using two of five public service systems) and a youth-nominated person most knowledgeable (PMK) [53].

Our findings align with evidence that point to this multi-agency work being about cultural transformation over more transactional, service–, or cohort focused approaches, with one international literature review highlighting the difference in ‘care philosophies’ between children mental health (developmental approach, involving families and nurturing) and adult mental health (clinical/diagnosis-focus, emphasis on client autonomy and individual responsibility) [54], and another report from Belgium around the challenge of commissioning standardised integrated service provision while acknowledging the need for bespoke solutions [55]. While focusing on risk management across children vs adult statutes, attempts to bridge the divide include propositions of ‘transitional safeguarding’ approaches that advocate for relational, developmental and contextual approaches [56].

## Conclusion

The policy landscape in the UK is shifting with a refresh of the child poverty strategy due, including crack downs on private sector profiteering, and addressing the 12.6% of young people aged 16 to 24 years currently not in education, employment or training (NEET) [57]. Some of this may be informed by emerging longitudinal evidence of early and preventative intervention through ‘Sure Start’ centres in the UK, published in October 2024 [58]. As with the wider direct and indirect cost of child poverty referenced above [5], we are well advised to consider the false economy of not seeking to prevent social and multiple adversity over the life course through assertive outreach and trusted professional styles of key-working.

What our findings suggest is that the shared intention between trusted professionals and young people is meeting a yearning for love and acceptance with meaningful connection (rather than rejection and closed doors), and that the third sector is uniquely placed to remind us of this.

The TP and similar approaches are happening locally and nationally, and ours is adding to this through a cross-sector, whole-systems lens and in the transitions space. They are ripe for spread and scale. Our Integrated Care Case serves as one example of preventative, proactive approaches to care coordination and continuity between care settings in one system. Innovative investment into academia-practice partnerships like the HDRCs that nourish the unique contributions of the third sector and lived experience might show a way. It would help if anchor institutions within Integrated Care Systems recognise the value and shared civic duty (https://haln.org.uk/case-studies/researchers-in-residence; accessed 27.05.25). These are crucial for a paradigm shift in applied integrated care research and to make learning portable and transferable (as opposed to generalisable [33]). This paper serves as a small example of embedding knowledge and evidence to support integrated care practice in real-time and at all levels of design and delivery.

## Additional Files

The additional files for this article can be found as follows:

10.5334/ijic.9055.s1 Changing Futures. YT Youth Service Factfinding.

10.5334/ijic.9055.s2 Your Story. Interview Protocol.

## Appendix A: ICC2442 Trusted Professional third sector reference list

1. Full list of references with hyperlinks in the main manuscript:

- John Seddon and Brendan O’Donovan. The Vanguard Method. *Systems Thinking*. 2023. Vol. 3:1-12. DOI: 10.54120/jost.000006
- Rosenhead J, Franco LA, Grint K, Friedland B. Complexity theory and leadership practice: A review, a critique, and some recommendations. The Leadership Quarterly. 2019;30(5):101304. https://doi.org/10.1016/j.leaqua.2019.07.002.
- Department for Education. Statutory guidance – Working together to safeguard children: Statutory guidance on multi-agency working to help, protect and promote the welfare of children.: HM Government; 2024; Available from: https://www.gov.uk/government/publications/working-together-to-safeguard-children--2.
- YoungMinds. Someone to turn to: Being a trusted adult for young people London: UK Youth; 2021; Available from:https://www.youngminds.org.uk/professional/community-support/someone-to-turn-to/.
- Golden Key Bristol UK; https://www.goldenkeybristol.org.uk/what-are-we-learning, accessed 27.05.25
- StBasils. Positive Pathway Framework: Preventing Youth Homelessness and Promoting Positive Transitions Birmingham: StBasils; 2020 [Available from: https://stbasils.org.uk/wp-content/uploads/2020/01/Final-framework1_PositivePathway_A4.pdf.
- The Homeless Link National Practice Team in partnership with Inspire Chilli and Expert Link. Becoming strengths-based: Overview of key ideas and principles: Homeless Link; [Available from: https://homelesslink-1b54.kxcdn.com/media/documents/Becoming_strengths-based.pdf.
- West Midlands Combined Authority. Punishing Abuse: Children in the West Midlands Criminal Justice System Manchester: West Midlands Combined Authority and the West Midlands Police and Crime Commissioner.; 2021 [Available from: https://www.wmca.org.uk/media/4678/punishing-abuse.pdf.
- Barnes M, Green R, Ross A. Understanding vulnerable young people: Analysis from the Longitudinal Study of Young People in England London: Department for Education; 2011 [Available from: https://assets.publishing.service.gov.uk/government/uploads/system/uploads/attachment_data/file/182672/DFE-RR118.pdf.
- Child Welfare Information Gateway. Prioritizing Youth Voice: The Importance of Authentic Youth Engagement in Case Planning: Department of Health and Human Services, Administration for Children and Families, Children’s Bureau.; 2021 [Available from: https://cwlibrary.childwelfare.gov/discovery/delivery/01CWIG_INST:01CWIG/1218769330007651

Below is a list of evidence and guidance that informed the work in early stages:

- Plymouth Safeguarding Children Partnership – Ten Wishes Charter: https://plymouthscb.co.uk/the-ten-wishes/
- Making Every Adult Matter (MEAM) Collective; https://meam.org.uk/the-meam-approach/#:~:text=The%20MEAM%20Approach%20helps%20local%20areas%20design%20and,voluntary%20agencies%20in%2050%20local%20areas%20across%20England.
- The Donella Meadows Project, Academy for Systems Change: “Leverage Points: Places to Intervene in a System”; Available from: https://donellameadows.org/archives/leverage-points-places-to-intervene-in-a-system/
- Youth Access UK; the national membership organisation for youth advice and counselling services across the UK: “A proven early intervention model”; Available from: https://www.youthaccess.org.uk/publications/policy/proven-early-intervention-model#:~:text=A%20report%20providing%20all%20the%20information%20you%20need,a%20range%20of%20health%20and%20social%20policy%20imperatives.
- Marmot, M. Fair society, healthy lives: the Marmot Review: strategic review of health inequalities in England post-2010. (2010) ISBN 9780956487001; Available from: https://www.gov.uk/research-for-development-outputs/fair-society-healthy-lives-the-marmot-review-strategic-review-of-health-inequalities-in-england-post-2010
- West Midlands Police and Crime Commissioner; 12^th^ March 2021; Available from: https://www.westmidlands-pcc.gov.uk/ground-breaking-report-evidence-shows-too-many-young-people-in-the-criminal-justice-system-suffer-from-violence-poverty-and-abuse-growing-up/

The first author also looked to (inter-)national literature early in this role to try and understand the problem for guidance on how to make up their job. Below are documents read in late 2022 when this project first started.

- Wood D, Crapnell T, Lau L, et al. Emerging Adulthood as a Critical Stage in the Life Course. 2017 Nov 21. In: Halfon N, Forrest CB, Lerner RM, et al., editors. Handbook of Life Course Health Development [Internet]. Cham (CH): Springer; 2018. Available from: https://www.ncbi.nlm.nih.gov/books/NBK543712/ doi: 10.1007/978-3-319-47143-3_7
- Ungar, M., Liebenberg, L., & Ikeda, J. (2014). Young People with Complex Needs: Designing Coordinated Interventions to Promote Resilience across Child Welfare, Juvenile Corrections, Mental Health and Education Services. *The British Journal of Social Work, 44*(3), 675–693. http://www.jstor.org/stable/43687664
- Just Lead Washington Leadership Academy; Flipping the iceberg model; Available from: https://justleadwa.org/wp-content/uploads/2018/09/REJI-Organizational-Toolkit_Tool-E.pdf
