# Supplementary material for: Trusted Professional Multi-Agency Transitions for Young People Facing Multiple Disadvantage – Learning from Co-Production by a Third Sector Partner in the Plymouth Alliance, UK
Source: Int J Integr Care. 2025 Jul 21;25(3):12. doi: 10.5334/ijic.9055 (PMC12292045; doi:10.5334/ijic.9055)
Supplement: Changing Futures. — YT Youth Service Factfinding. [file ijic-25-3-9055-s1.pdf]

Changing Futures – YT - Youth Service Factfinding

Organization name: Do you use **Manta?:**

Contact spoken to:

Trauma informed network member: Yes/No

Any other networks:

Service Name:

Do you support ages: 16/17: Yes/No **18+:** Yes/No

What is the remit of Service:

Additional Services provided beyond service remit:

Is there a model used for the service, if yes what is it?:

Estimated percentage of individuals who use service with Multiple Disadvantage of 3 or more (Homeless, Substance misuse, Mental Health, Domestic Abuse, CJS):

Actual age group supported: Number of **Clients/ Max capacity:**

is there a **Waiting List/how long:** **Who funds the service:**

do you feel your service is meeting demand: Yes/No **Time limit for support:**

is the service Face to face?: are there **Service exclusions/ what are they:**

What would the service consider a Successful Outcome:

How are your outcomes measured/ reported to funders?:

Positives of the current way of working (system as a whole or individual service):

Negatives of the current way of working:

Gaps Identified within service or with regards to youth transitions across the systems:

Are there any risks to service delivery:

Do you have a Youth Transition worker or equivalent?:

Do you have any **partner organizations:**

Is there any support needed for your service:

Core Issues from this discussion:

<u>Additional</u>

Gaps in support area beyond individuals?

Women at risk – is there any additional provision?

Are there any others at risk locally from your perspective?

Potential Case Study of successful or unsuccessful transition from youth to adult services:

<u>Demo details</u>

Current 16/17:

%:

Male:

Female:

Trans/Non-bin/other:

Plymouth Residents: Non-Plymouth:

Care leavers: Non-Care leavers:

Current 18-25:

%:

Male:

Female:

Trans/Non-bin/other:

Plymouth Residents: Non-Plymouth:

Care leavers: Non-Care leavers:
