# Supplementary material for: Trusted Professional Multi-Agency Transitions for Young People Facing Multiple Disadvantage – Learning from Co-Production by a Third Sector Partner in the Plymouth Alliance, UK
Source: Int J Integr Care. 2025 Jul 21;25(3):12. doi: 10.5334/ijic.9055 (PMC12292045; doi:10.5334/ijic.9055)
Supplement: Your Story. — Interview Protocol. [file ijic-25-3-9055-s2.pdf]

Whats currently going on for you and when did this start?
1Why do you find yourself here?
1Where do you currently live?
1Who do you currently live with?
1When did your situation start?
1What is your situation?
1Whats going on for you?

Who are you working with to resolve this and why?
2What are your options? What do you think your options are?
2Housing – what support are you hoping for?
2Who are you working with?
2When do you hope to be housed?/Where do you hope to be housed?
2What are your next steps?
2What do you know about the housing in Plymouth?
2Where did you go to get information on housing?
2Signpost/warm handover
2Where have you been for support?
2How many places?

How is this impacting you?
3What is Plymouth like for you?
3How do you feel? What's it like to be you?
3How do you feel you've been supported?
3Who is your social support?
3How are you managing socially, emotionally etc?
3Is this different to what you think will happen?

What would you want other young people and services to know?
4What Matters to them?
4What do you think should happen?
4How many times re-talking story?
4Did they feel heard?
